# Current Challenges and Pitfalls in Soil Metagenomics

**DOI:** 10.3390/microorganisms10101900

**Published:** 2022-09-25

**Authors:** Marcio F. A. Leite, Sarah W. E. B. van den Broek, Eiko E. Kuramae

**Affiliations:** 1 Department of Microbial Ecology, Netherlands Institute of Ecology, 6708 PB Wageningen, The Netherlands; 2 Ecology and Biodiversity, Institute of Environmental Biology, Utrecht University, 3584 CS Utrecht, The Netherlands

**Keywords:** soil physicochemical properties, microbial ecology, metagenomics, untreated controls, data-sharing guidelines

## Abstract

Soil microbial communities are essential components of agroecological ecosystems that influence soil fertility, nutrient turnover, and plant productivity. Metagenomics data are increasingly easy to obtain, but studies of soil metagenomics face three key challenges: (1) accounting for soil physicochemical properties; (2) incorporating untreated controls; and (3) sharing data. Accounting for soil physicochemical properties is crucial for better understanding the changes in soil microbial community composition, mechanisms, and abundance. Untreated controls provide a good baseline to measure changes in soil microbial communities and separate treatment effects from random effects. Sharing data increases reproducibility and enables meta-analyses, which are important for investigating overall effects. To overcome these challenges, we suggest establishing standard guidelines for the design of experiments for studying soil metagenomics. Addressing these challenges will promote a better understanding of soil microbial community composition and function, which we can exploit to enhance soil quality, health, and fertility.

## 1. Introduction

Soil microbial communities in agroecosystems contribute to (i) soil health, nutrient cycling, and fertility [1,2], thereby increasing pathogen suppression [3,4] and processes that enhance crop yields [5,6]; (ii) the production [7,8] and mitigation of greenhouse gas (GHG) emissions [9]; and (iii) carbon sequestration through the formation of mineral-associated organic matter [2,10] and carbon storage [11,12]. All these soil microbe functions are directly or indirectly linked to soil physicochemical properties.

The advent of next generation sequencing (NGS) has enabled in-depth investigations of the composition and functions of whole microbial communities [13,14] and allowed the performance of metagenomic studies [15]. The term metagenomics was coined in 1998 by Handelsman et al. [16] and refers to the genomic information of the microbial community inhabiting an environment. The decreasing cost of NGS has facilitated the analysis of complex environments and has allowed researchers to perform metagenomic studies on soil [17] and produce large-scale data. However, the ease of generating metagenomic data brings new challenges for study design and analysis. First, soil microbial communities and their functions are influenced by environmental factors such as soil physicochemical properties, but this complexity of the soil microbiome is largely neglected [18,19]. Second, proper controls are necessary to better understand the influence of the environment on soil microbial communities. Third, large, published, and publicly available datasets are necessary to conduct meta-analyses to develop a better understanding of microbial communities on a larger scale. In this paper, we discuss how these three challenges are limiting further development of the field of metagenomics and how they can be overcome. Moreover, we outline the implications of surmounting these obstacles for our understanding of soil microbial communities.

## 2. Soil Physicochemical Properties Improves Our Understanding of Community Processes in Soil Microbiomes

Soil physicochemical properties play a crucial role in determining soil microbiome composition and function [20,21]. However, most soil microbiome studies either do not include soil physicochemical properties or limit measurements to pH, soil organic carbon (SOC), total nitrogen (TN), total carbon (TC), moisture, and/or temperature, which do not fully reflect the complex soil chemical matrix and its various constituent elements (e.g., N, C, K, P, Zn, Fe, Ca, Mn, Mg). Soil properties vary with soil depth [22], as shown for SOC, total N [23,24], and total P [25]. Moreover, soil properties, particularly pH, are dynamic and fluctuate with changes in climate [26], environment [27], and the aboveground population [28].

A common pitfall of soil microbiome studies is a lack of measurements of soil physicochemical properties. This is especially important in agricultural studies because land use determines soil physiochemical properties [20], which change nutrient availability and cycling [2]. These changes explain (part of) the shifts observed in soil microbial communities, root–soil interactions [29], pathogen suppression [30], and microbial processes [31]. If these properties are not measured before the start of a long-term experiment, the control cannot be used to investigate the influence of time on soil physicochemical properties because time changes soil physicochemical properties [32]. In other words, the direct effect of the treatment cannot be separated from the indirect effect of changes in soil physicochemical properties.

Including soil physicochemical properties in every soil metagenomic study does not only ease the comparison of studies, but also increases the knowledge within a system. This will allow us to further disentangle soil microbial communities by linking specific microorganisms or functions to precise chemical changes [33]. Microbial responses are context-dependent; they change with soil disturbance [34], climate, and nutrient availability [26,35]. Nutrients also determine the abundance of root promoting microorganisms [36], which can even help explain changes in plant productivity. These contexts change not only the content of microbial communities but also their interactions [37]. The choice to not include physicochemical properties in a soil microbial community study means there is a vital part missing in explaining soil microbial community responses.

In addition, accounting for soil properties can provide a better understanding of why specific microbes are especially sensitive to certain abiotic changes and the potential impact of this sensitivity on their ecosystem function. Recently, Leite et al. [36] showed that the efficiency of plant-growth-promoting microbes depends on the availability of nitrogen. A stronger grasp of the effects of soil physicochemical properties on soil microbial communities will provide insights into how these microbial communities are shaped and their roles in soil quality, health, and plant productivity.

## 3. Untreated Controls Are Critical for a Better Understanding of Soil Microbial Communities

Soil microbial communities are complex and susceptible to change [5,19]; this emphasizes the importance of establishing a baseline for comparison with the targeted treatment. Untreated controls provide such a baseline for determining whether a change in the soil microbial community is due to the treatment or to an unknown factor (e.g., stochastic processes). To illustrate this need, consider a hypothetical experiment comparing organic amendment and inorganic fertilizer; in such a comparison, the abundance of Acidobacteria is high in the treatment with organic amendment but low in the treatment with inorganic fertilizer. These results have two potential explanations: (1) organic amendment increases the abundance of Acidobacteria, and (2) inorganic fertilizers decrease the abundance of Acidobacteria. In the absence of a control containing the original soil without any amendment (untreated), both explanations are valid. Thus, including a control simplifies the interpretation of the results and facilitates the design of follow-up experiments.

Nonetheless, most comparisons of organic amendments with inorganic fertilizers directly compare the microbial communities in the two treatments. This approach does not provide a clear picture of which treatment is the main cause of microbial shifts. One reason for the lack of untreated controls is that soil biology agricultural research is often conducted on farms, where not treating an area of soil will have economic and food-security consequences if crop yields are reduced. A second reason is that expanding the number of samples to include a control increases the cost as well as the burden of data analysis, which may be an issue when there are time constraints. A potential solution is to maintain a small area of the plot without any fertilizers for use as a control, which would benefit research without reducing crop yield. If there are time or cost constraints, a single plot could be kept free of any treatments instead of replicating the control three or more times. A third reason is in cases where the area is highly farmed, so there is no untreated land available. Even when a piece of land is untreated from that time, the history of the land can still influence and explain changes in the soil microbial community [38]. An untreated control is also important when accounting for the influence of soil physicochemical properties. Without an untreated control, it is very difficult to determine whether changes in the soil microbial community reflect soil physicochemical properties, time, or the treatment. It is important to realize that the lack of an untreated control impacts the vision on the soil microbial community. The lack of an untreated control may mislead the conclusions. For instance, Soman et al. [39] found that bacterial diversity was higher in soil with poultry litter than with inorganic fertilizer, it is the case that poultry litter is better for bacterial diversity than inorganic fertilizer. However, the untreated control shows that bacterial diversity was higher without treatment. Thus, inorganic fertilizer and poultry litter both lower bacterial diversity and it might be better to use no fertilizer if an increase in bacterial diversity is the aim.

Some researchers have developed creative solutions for including a control sample. One common approach is to measure the soil microbial communities and/or soil physicochemical properties before the start of the study for use as a control. However, this approach neglects the possibility of changes in the soil microbial community and/or soil physicochemical properties over time, especially in long-term field experiments [40,41]. Consequently, it is important to have a real-time control that is exposed to the elements in the same way as the treatments. Another common solution is the use of inorganic fertilizer as a control. However, inorganic fertilizer also changes the soil microbial community and soil physicochemical properties [42], making it impossible to establish a baseline. Other researchers use non-agricultural fields, such as grassland, as controls, but such controls cannot be reliably compared to agricultural fields because soil microbial communities differ greatly among different land-use systems [43]. Absolute microbiome profiling quantifies absolute abundance in metagenome samples which eases comparisons between soil microbial communities [37]. This does not, however, lift the need for an untreated control; it does not explain which environmental variables influenced the soil microbial community during the experiment, or how they had this influence.

Including an untreated control in every soil microbiome study would contribute to reproducibility and facilitate the integration of studies in meta-analyses. In addition, the effects of the treatments could be separated from environmental effects. Widespread adoption of untreated controls in soil microbial community research would also enable the creation of a database of microbial baselines that could be used to infer possible consequences of changes in soil microbial communities.

## 4. FAIR Data Are Needed to Better Understand the Soil Microbiome

More sequencing data are being generated than ever before [44,45], which has increased the complexity of conducting transparent and reproducible research. Data sharing and transparency are key factors for ensuring reproducibility and comparability between studies. To help structure data management and ensure reproducibility in scientific research, the FAIR (findable, accessible, interoperable, reusable) guidelines for scientific data were introduced in 2016 [46]. This approach has since been evaluated by the biomedical research community [47,48] and has also been proposed for environmental metagenomics [49]. Many initiatives have improved data sharing and standardization, such as TerraGenome [50], the Earth Microbiome Project [51,52], and the Genomics Standards Consortium [53].

There is an increasing need for meta-analyses of the soil microbiome to uncover soil microbial community mechanisms and untangle the influences of soil type, location, soil physicochemical properties, and treatments. However, the FAIR approach is not widely used in soil metagenomics research. For instance, if the FAIR approach is not applied, information such as soil depth is missing from the methods. This loss of information reduces reproducibility and increases the difficulty of metagenomic meta-analyses. Data are not always checked for metadata correctness and completeness. This could be improved with guidelines to standardize metadata across platforms [54].

The FAIR principles should be prioritized from the start of the project as they require mutual awareness and consent from the relevant group members and a consideration of costs involved in maintaining FAIR data [55]. Reasons for not applying the FAIR principles in environmental metagenomics include work regulations preventing data sharing, technical difficulties in sharing data [56], or underestimation of the importance of data sharing [57]. One option for addressing these issues is to include data-sharing seminars or lectures in PhD programs and conferences to teach prospective researchers about the importance of data sharing. Another option is to develop data-sharing tools that facilitate data sharing [56]. Data-sharing requirements are a solution that has already been implemented by funding agencies which has significantly improved data sharing over the last years [58]. Despite these initiatives, some data-sharing problems have remained, because the guidelines for data sharing as given by a journal are not always translated to reality. Vasilevsky et al. [59] found that encouragement of data sharing is not pursued by journals as they do not require it. They found that, for journals requiring data sharing, the lack of data does not stop them from publishing a paper. Some journals require that a data statement is included, but authors are often not compliant to this statement [60] or the data are not findable [61]. Another workaround that some authors use to fulfil the data-sharing requirements by a journal, is to only publish part of their sequencing data [58]. Following FAIR guidelines with software [45] and R packages [62] is also important to create an open-data environment. Stricter journal guidelines with specific guidance increases FAIR data management and data sharing [59,63].

Applying the FAIR principle to all studies of soil metagenomics will allow meta-analyses to elucidate the dynamics of soil microbial communities and provide new insights into published data. It would also provide greater transparency, which would allow us to learn more about soil microbial communities and how they are affected by environmental conditions. Most importantly, we could work together as a scientific community to find solutions and advance the development of the field of metagenomics.

## 5. Discussion

Soil microbial communities are a vital part of our ecosystems that contribute to disease suppression, nutrient cycling, and soil fertility. The emerging field of metagenomics has the potential to uncover the functions and mechanisms of soil microbial communities. However, common guidelines are necessary to ensure comparability between studies and a better understanding of soil microbial communities. We argue that those guidelines should start with (i) a more detailed characterization of soil properties, (ii) the inclusion of untreated controls to avoid biased conclusions on shifts of community structure, and (iii) data-sharing and transparency measures to ensure reproducibility.

Many soil physicochemical properties affect soil microbial communities, and if we do not include at least some soil physicochemical properties, we will be navigating in the dark. The importance of including soil physicochemical properties goes beyond cross-study comparisons and is essential to disentangle soil microbial community mechanisms and connect them to specific chemical changes. Accounting for the soil physicochemical properties also brings the additional benefit of indirectly showing the effect of other soil organisms that shape the soil factors (e.g., decomposers [64]) without the need to characterize them. This will contribute to a better understanding of the role of soil physicochemical properties and the specific microorganisms that we can use for precision farming and increasing soil health and plant productivity. It is especially important to include soil physicochemical properties in tropical soil, where soil properties may be very different to temperature soils [65], which can change soil microbial communities significantly [5].

Adopting appropriate controls will promote reproducibility in metagenomics and establish a clear microbial baseline for soils. In-field and real-time controls can be used to create a database of soil microbial community baselines around the globe, which we can combine with soil physicochemical property data to better understand soil changes [66,67,68]. However, the creation of a true soil microbial community baseline demands a renewed emphasis on transparent data sharing. Soil metagenomics is complex, and the results are influenced by many factors, including the methods of DNA isolation, sequencing, and data analysis [69,70,71]. Therefore, it is essential not only to share our data in a FAIR way but also to include FAIR metadata [46,48,72].

We present the three key challenges, but we also acknowledge the existence of many other challenges. Mocali and Benedetti [73] highlighted that, in order to study soil microbial communities via soil metagenomics, we need to consider efficient DNA-extraction methods with well-defined screening strategies and sequencing approaches. For example, Dimitrov et al. [69] proposed that successive DNA extractions optimize the DNA yield and led to a better understanding of the microbial community composition. Altogether, we highlight that we need also to consider methodological challenges to fully embrace the complexity of soil microbial community.

Prosser [74] argued that microbial ecology research should go beyond describing the microbial community and its functionality. We extend this recommendation by suggesting common guidelines to ensure that data collection efforts are not wasted or repeated unnecessarily. These challenges are also applicable to metatranscriptomics, metabolomics, and metaproteomics [75]. However, these disciplines are largely underexplored for soil microbial communities due to operational challenges [76]. The challenges motivating these guidelines need to be solved to ensure that the field of soil metagenomics continues to expand. Strengthening our research and increasing our understanding of the soil microbiome will accelerate efforts to tackle issues related to carbon sequestration, greenhouse gas mitigation, and food security.

## 6. Conclusions

In this paper, we discuss the three critical challenges faced by soil metagenomics research: (1) accounting for soil physicochemical properties; (2) incorporating untreated controls; and (3) sharing data. We suggest resolving these issues by establishing standard guidelines for experimental design in soil metagenomics. A strict procedure by journals and funding agencies will help to convince researchers to adhere to these guidelines. Overcoming these challenges benefits the field by the increased understanding of soil microbial community responses and facilitating cross-study analyses such as meta-analyses and global models.

## Data Availability

Not applicable.

## References

[1] Chaparro J.M., Sheflin A.M., Manter D.K., Vivanco J.M. (2012). Manipulating the Soil Microbiome to Increase Soil Health and Plant Fertility. Biol. Fertil. Soils.

[2] Bossolani J.W., Crusciol C.A.C., Leite M.F.A., Merloti L.F., Moretti L.G., Pascoaloto I.M., Kuramae E.E. (2021). Modulation of the Soil Microbiome by Long-Term Ca-Based Soil Amendments Boosts Soil Organic Carbon and Physicochemical Quality in a Tropical No-till Crop Rotation System. Soil Biol. Biochem..

[3] Mendes R., Garbeva P., Raaijmakers J.M. (2013). The Rhizosphere Microbiome: Significance of Plant Beneficial, Plant Pathogenic, and Human Pathogenic Microorganisms. FEMS Microbiol. Rev..

[4] Latz E., Eisenhauer N., Rall B.C., Scheu S., Jousset A. (2016). Unravelling Linkages between Plant Community Composition and the Pathogen-Suppressive Potential of Soils. Sci. Rep..

[5] Trivedi P., Delgado-Baquerizo M., Anderson I.C., Singh B.K. (2016). Response of Soil Properties and Microbial Communities to Agriculture: Implications for Primary Productivity and Soil Health Indicators. Front. Plant Sci..

[6] Momesso L., Crusciol C.A.C., Leite M.F.A., Bossolani J.W., Kuramae E.E. (2022). Forage Grasses Steer Soil Nitrogen Processes, Microbial Populations, and Microbiome Composition in A Long-Term Tropical Agriculture System. Agric. Ecosyst. Environ..

[7] Lourenço K.S., Suleiman A.K., Pijl A., Van Veen J.A., Cantarella H., Kuramae E.E. (2018). Resilience of the Resident Soil Microbiome to Organic and Inorganic Amendment Disturbances and to Temporary Bacterial Invasion. Microbiome.

[8] Lourenço K.S., Costa O.Y.d.A., Cantarella H., Kuramae E.E. (2022). Ammonia-Oxidizing Bacteria and Fungal Denitrifier Diversity Are Associated with N_2_O Production in Tropical Soils. Soil Biol. Biochem..

[9] Malyan S.K., Bhatia A., Tomer R., Harit R.C., Jain N., Bhowmik A., Kaushik R. (2021). Mitigation of Yield-Scaled Greenhouse Gas Emissions from Irrigated Rice through Azolla, Blue-Green Algae, and Plant Growth–Promoting Bacteria. Environ. Sci. Pollut. Res..

[10] Fontaine S., Hénault C., Aamor A., Bdioui N., Bloor J.M.G., Maire V., Mary B., Revaillot S., Maron P.-A. (2011). Fungi Mediate Long Term Sequestration of Carbon and Nitrogen in Soil through Their Priming Effect. Soil Biol. Biochem..

[11] Liang C., Schimel J.P., Jastrow J.D. (2017). The Importance of Anabolism in Microbial Control over Soil Carbon Storage. Nat. Microbiol..

[12] Liang C., Amelung W., Lehmann J., Kästner M. (2019). Quantitative Assessment of Microbial Necromass Contribution to Soil Organic Matter. Glob. Change Biol..

[13] Nkongolo K.K., Narendrula-Kotha R. (2020). Advances in Monitoring Soil Microbial Community Dynamic and Function. J. Appl. Genet..

[14] Breitkreuz C., Heintz-Buschart A., Buscot F., Wahdan S.F.M., Tarkka M., Reitz T. (2021). Can We Estimate Functionality of Soil Microbial Communities from Structure-Derived Predictions? A Reality Test in Agricultural Soils. Microbiol. Spectr..

[15] Escobar-Zepeda A., Vera-Ponce de León A., Sanchez-Flores A. (2015). The Road to Metagenomics: From Microbiology to DNA Sequencing Technologies and Bioinformatics. Front. Genet..

[16] Handelsman J., Rondon M.R., Brady S.F., Clardy J., Goodman R.M. (1998). Molecular Biological Access to the Chemistry of Unknown Soil Microbes: A New Frontier for Natural Products. Chem. Biol..

[17] Daniel R. (2005). The Metagenomics of Soil. Nat. Rev. Microbiol..

[18] Guseva K., Darcy S., Simon E., Alteio L.V., Montesinos-Navarro A., Kaiser C. (2022). From Diversity to Complexity: Microbial Networks in Soils. Soil Biol. Biochem..

[19] Kuramae E.E., Yergeau E., Wong L.C., Pijl A.S., Veen J.A., Kowalchuk G.A. (2012). Soil Characteristics More Strongly Influence Soil Bacterial Communities than Land-use Type. FEMS Microbiol. Ecol..

[20] Liu C., Jin Y., Hu Y., Tang J., Xiong Q., Xu M., Bibi F., Beng K.C. (2019). Drivers of Soil Bacterial Community Structure and Diversity in Tropical Agroforestry Systems. Agric. Ecosyst. Environ..

[21] Dunn L., Lang C., Marilleau N., Terrat S., Biju-Duval L., Lelièvre M., Perrin S., Chemidlin Prévost-Bouré N. (2021). Soil Microbial Communities in the Face of Changing Farming Practices: A Case Study in an Agricultural Landscape in France. PLoS ONE.

[22] Naylor D., McClure R., Jansson J. (2022). Trends in Microbial Community Composition and Function by Soil Depth. Microorganisms.

[23] Yu H., Zha T., Zhang X., Ma L. (2019). Vertical Distribution and Influencing Factors of Soil Organic Carbon in the Loess Plateau, China. Sci. Total Environ..

[24] Qi Q., Zhang D., Zhang M., Tong S., Wang W., An Y. (2021). Spatial Distribution of Soil Organic Carbon and Total Nitrogen in Disturbed Carex Tussock Wetland. Ecol. Indic..

[25] Bai Y., Chen S., Shi S., Qi M., Liu X., Wang H., Wang Y., Jiang C. (2020). Effects of Different Management Approaches on the Stoichiometric Characteristics of Soil C, N, and P in a Mature Chinese Fir Plantation. Sci. Total Environ..

[26] Shi Y., Su C., Wang M., Liu X., Liang C., Zhao L., Zhang X., Minggagud H., Feng G., Ma W. (2020). Modern Climate and Soil Properties Explain Functional Structure Better Than Phylogenetic Structure of Plant Communities in Northern China. Front. Ecol. Evol..

[27] Bahram M., Hildebrand F., Forslund S.K., Anderson J.L., Soudzilovskaia N.A., Bodegom P.M., Bengtsson-Palme J., Anslan S., Coelho L.P., Harend H. (2018). Structure and Function of the Global Topsoil Microbiome. Nature.

[28] Zhao M., Sun M., Xiong T., Tian S., Liu S. (2022). On the Link between Tree Size and Ecosystem Carbon Sequestration Capacity across Continental Forests. Ecosphere.

[29] Bulgarelli R.G., Leite M.F.A., de Hollander M., Mazzafera P., Andrade S.A.L., Kuramae E.E. (2022). Eucalypt Species Drive Rhizosphere Bacterial and Fungal Community Assembly but Soil Phosphorus Availability Rearranges the Microbiome. Sci. Total Environ..

[30] Su L., Feng H., Mo X., Sun J., Qiu P., Liu Y., Zhang R., Kuramae E.E., Shen B., Shen Q. (2022). Potassium Phosphite Enhanced the Suppressive Capacity of the Soil Microbiome against the Tomato Pathogen Ralstonia Solanacearum. Biol. Fertil. Soils.

[31] Reichenbach M., Fiener P., Garland G., Griepentrog M., Six J., Doetterl S. (2021). The Role of Geochemistry in Organic Carbon Stabilization against Microbial Decomposition in Tropical Rainforest Soils. SOIL.

[32] Cassman N.A., Leite M.F., Pan Y., de Hollander M., van Veen J.A., Kuramae E.E. (2016). Plant and Soil Fungal but Not Soil Bacterial Communities Are Linked in Long-Term Fertilized Grassland. Sci. Rep..

[33] Saghaï A., Banjeree S., Degrune F., Edlinger A., García-Palacios P., Garland G., Heijden M.G.A., Herzog C., Maestre F.T., Pescador D.S. (2022). Diversity of Archaea and Niche Preferences among Putative Ammonia-oxidizing Nitrososphaeria Dominating across European Arable Soils. Environ. Microbiol..

[34] Seitz T.J., Schütte U.M.E., Drown D.M. (2022). Unearthing Shifts in Microbial Communities Across a Soil Disturbance Gradient. Front. Microbiol..

[35] She W., Bai Y., Zhang Y., Qin S., Feng W., Sun Y., Zheng J., Wu B. (2018). Resource Availability Drives Responses of Soil Microbial Communities to Short-Term Precipitation and Nitrogen Addition in a Desert Shrubland. Front. Microbiol..

[36] Leite M.F.A., Dimitrov M.R., Freitas-Iório R.P., de Hollander M., Cipriano M.A.P., Andrade S.A.L., da Silveira A.P.D., Kuramae E.E. (2021). Rearranging the Sugarcane Holobiont via Plant Growth-Promoting Bacteria and Nitrogen Input. Sci. Total Environ..

[37] Zhou L., Guan D., Yuan X., Zhang M., Gao W. (2021). Quantifying the Spatiotemporal Characteristics of Ecosystem Services and Livelihoods in China’s Poverty-Stricken Counties. Front. Earth Sci..

[38] Schmidt J.E., Vannette R.L., Igwe A., Blundell R., Casteel C.L., Gaudin A.C. (2019). Effects of Agricultural Management on Rhizosphere Microbial Structure and Function in Processing Tomato. Appl. Environ. Microbiol..

[39] Celestina C., Wood J.L., Manson J.B., Wang X., Sale P.W., Tang C., Franks A.E. (2019). Microbial Communities in Top- and Subsoil of Repacked Soil Columns Respond Differently to Amendments but Their Diversity Is Negatively Correlated with Plant Productivity. Sci. Rep..

[40] Kuramae E.E., Gamper H.A., Yergeau E., Piceno Y.M., Brodie E.L., DeSantis T.Z., Andersen G.L., van Veen J.A., Kowalchuk G.A. (2010). Microbial Secondary Succession in a Chronosequence of Chalk Grasslands. ISME J..

[41] Pan Y., Cassman N., de Hollander M., Mendes L.W., Korevaar H., Geerts R.H., van Veen J.A., Kuramae E.E. (2014). Impact of Long-Term N, P, K, and NPK Fertilization on the Composition and Potential Functions of the Bacterial Community in Grassland Soil. FEMS Microbiol. Ecol..

[42] Soman C., Li D., Wander M.M., Kent A.D. (2017). Long-Term Fertilizer and Crop-Rotation Treatments Differentially Affect Soil Bacterial Community Structure. Plant Soil.

[43] Kuramae E.E., Zhou J.Z., Kowalchuk G.A., van Veen J.A. (2014). Soil-Borne Microbial Functional Structure across Different Land Uses. Sci. World J..

[44] Land M., Hauser L., Jun S.-R., Nookaew I., Leuze M.R., Ahn T.-H., Karpinets T., Lund O., Kora G., Wassenaar T. (2015). Insights from 20 Years of Bacterial Genome Sequencing. Funct. Integr. Genom..

[45] Katz K., Shutov O., Lapoint R., Kimelman M., Brister J.R., O’Sullivan C. (2022). The Sequence Read Archive: A Decade More of Explosive Growth. Nucleic Acids Res..

[46] Wilkinson M.D., Dumontier M., Aalbersberg I.J., Appleton G., Axton M., Baak A., Blomberg N., Boiten J.-W., da Silva Santos L.B., Bourne P.E. (2016). The FAIR Guiding Principles for Scientific Data Management and Stewardship. Sci. Data.

[47] Boeckhout M., Zielhuis G.A., Bredenoord A.L. (2018). The FAIR Guiding Principles for Data Stewardship: Fair Enough?. Eur. J. Hum. Genet..

[48] Madduri R., Chard K., D’Arcy M., Jung S.C., Rodriguez A., Sulakhe D., Deutsch E., Funk C., Heavner B., Richards M. (2019). Reproducible Big Data Science: A Case Study in Continuous FAIRness. PLoS ONE.

[49] Ten Hoopen P., Finn R.D., Bongo L.A., Corre E., Fosso B., Meyer F., Mitchell A., Pelletier E., Pesole G., Santamaria M. (2017). The Metagenomic Data Life-Cycle: Standards and Best Practices. GigaScience.

[50] Vogel T.M., Simonet P., Jansson J.K., Hirsch P.R., Tiedje J.M., Van Elsas J.D., Bailey M.J., Nalin R., Philippot L. (2009). TerraGenome: A Consortium for the Sequencing of a Soil Metagenome. Nat. Rev. Microbiol..

[51] Thompson L.R., Sanders J.G., McDonald D., Amir A., Ladau J., Locey K.J., Prill R.J., Tripathi A., Gibbons S.M., Ackermann G. (2017). A Communal Catalogue Reveals Earth’s Multiscale Microbial Diversity. Nature.

[52] Gilbert J.A., Jansson J.K., Knight R. (2014). The Earth Microbiome Project: Successes and Aspirations. BMC Biol..

[53] Field D., Amaral-Zettler L., Cochrane G., Cole J.R., Dawyndt P., Garrity G.M., Gilbert J., Glöckner F.O., Hirschman L., Karsch-Mizrachi I. (2011). The Genomic Standards Consortium. PLoS Biol..

[54] Schoch C.L., Ciufo S., Domrachev M., Hotton C.L., Kannan S., Khovanskaya R., Leipe D., Mcveigh R., O’Neill K., Robbertse B. (2020). NCBI Taxonomy: A Comprehensive Update on Curation, Resources and Tools. Database.

[55] Koers H., Bangert D., Hermans E., van Horik R., de Jong M., Mokrane M. (2020). Recommendations for Services in a FAIR Data Ecosystem. Patterns.

[56] Hajduk G.K., Jamieson N.E., Baker B.L., Olesen O.F., Lang T. (2019). It Is Not Enough That We Require Data to Be Shared; We Have to Make Sharing Easy, Feasible and Accessible Too!. BMJ Glob. Health.

[57] Mardis E.R. (2016). The Challenges of Big Data. Dis. Model. Mech..

[58] Jurburg S.D., Konzack M., Eisenhauer N., Heintz-Buschart A. (2020). The Archives Are Half-Empty: An Assessment of the Availability of Microbial Community Sequencing Data. Commun. Biol..

[59] Vasilevsky N.A., Minnier J., Haendel M.A., Champieux R.E. (2017). Reproducible and Reusable Research: Are Journal Data Sharing Policies Meeting the Mark?. PeerJ.

[60] Gabelica M., Bojčić R., Puljak L. (2022). Many Researchers Were Not Compliant with Their Published Data Sharing Statement: A Mixed-Methods Study. J. Clin. Epidemiol..

[61] Federer L.M., Belter C.W., Joubert D.J., Livinski A., Lu Y.-L., Snyders L.N., Thompson H. (2018). Data Sharing in *PLoS ONE*: An Analysis of Data Availability Statements. PLoS ONE.

[62] Vuorre M., Crump M.J. (2021). Sharing and Organizing Research Products as R Packages. Behav. Res. Methods.

[63] Mayer G., Müller W., Schork K., Uszkoreit J., Weidemann A., Wittig U., Rey M., Quast C., Felden J., Glöckner F.O. (2021). Implementing FAIR Data Management within the German Network for Bioinformatics Infrastructure (de.NBI) Exemplified by Selected Use Cases. Brief. Bioinform..

[64] Hättenschwiler S., Tiunov A.V., Scheu S. (2005). Biodiversity and Litter Decomposition in Terrestrial Ecosystems. Annu. Rev. Ecol. Evol. Syst..

[65] Doetterl S., Asifiwe R.K., Baert G., Bamba F., Bauters M., Boeckx P., Bukombe B., Cadisch G., Cooper M., Cizungu L.N. (2021). Organic Matter Cycling along Geochemical, Geomorphic, and Disturbance Gradients in Forest and Cropland of the African Tropics—Project TropSOC Database Version 1.0. Earth Syst. Sci. Data.

[66] Manter D.K., Delgado J.A., Blackburn H.D., Harmel D., Pérez de León A.A., Honeycutt C.W. (2017). Why We Need a National Living Soil Repository. Proc. Natl. Acad. Sci. USA.

[67] Chu H., Gao G.-F., Ma Y., Fan K., Delgado-Baquerizo M. (2020). Soil Microbial Biogeography in a Changing World: Recent Advances and Future Perspectives. mSystems.

[68] Guerra C.A., Delgado-Baquerizo M., Duarte E., Marigliano O., Görgen C., Maestre F.T., Eisenhauer N. (2021). Global Projections of the Soil Microbiome in the Anthropocene. Glob. Ecol. Biogeogr..

[69] Dimitrov M.R., Veraart A.J., de Hollander M., Smidt H., van Veen J.A., Kuramae E.E. (2017). Successive DNA Extractions Improve Characterization of Soil Microbial Communities. PeerJ.

[70] Murali A., Bhargava A., Wright E.S. (2018). IDTAXA: A Novel Approach for Accurate Taxonomic Classification of Microbiome Sequences. Microbiome.

[71] Leite M.F.A., Kuramae E.E. (2020). You Must Choose, but Choose Wisely: Model-Based Approaches for Microbial Community Analysis. Soil Biol. Biochem..

[72] Nayfach S., Pollard K.S. (2016). Toward Accurate and Quantitative Comparative Metagenomics. Cell.

[73] Mocali S., Benedetti A. (2010). Exploring Research Frontiers in Microbiology: The Challenge of Metagenomics in Soil Microbiology. Res. Microbiol..

[74] Prosser J.I. (2020). Putting Science Back into Microbial Ecology: A Question of Approach. Philos. Trans. R. Soc. B Biol. Sci..

[75] Shakya M., Lo C.-C., Chain P.S.G. (2019). Advances and Challenges in Metatranscriptomic Analysis. Front. Genet..

[76] Djemiel C., Dequiedt S., Karimi B., Cottin A., Horrigue W., Bailly A., Boutaleb A., Sadet-Bourgeteau S., Maron P.-A., Chemidlin Prévost-Bouré N. (2022). Potential of Meta-Omics to Provide Modern Microbial Indicators for Monitoring Soil Quality and Securing Food Production. Front. Microbiol..

